# First detection of Candidatus *Rickettsia barbariae* in the flea *Vermipsylla alakurt* from north-western China

**DOI:** 10.1186/s13071-016-1614-2

**Published:** 2016-06-07

**Authors:** Shan-Shan Zhao, Hong-Yu Li, Xiao-Ping Yin, Zhi-Qiang Liu, Chuang-Fu Chen, Yuan-Zhi Wang

**Affiliations:** School of Medicine, Shihezi University, Shihezi, Xinjiang Uygur Autonomous Region 832000 China; Alashankou Entry-Exit Inspection and Quarantine Bureau, Alashankou, 833418 China; College of Animal Science and Technology, Shihezi University, Shihezi, 832000 China

**Keywords:** Candidatus *Rickettsia barbariae*, *Vermipsylla alakurt*, North-western China

## Abstract

**Background:**

*Vermipsylla* is a genus of the family Vermipsyllidae within the order Siphonaptera of fleas. *Vermipsylla alakurt* is mainly distributed in alpine pastoral areas of Kazakhstan, Mongolia, China and Nepal, and infests sheep, yaks and horses, causing irritation, poor condition, anaemia and even death. However, to date, no rickettsial agents have been reported in *V. alakurt*.

**Findings:**

A total of 133 fleas were collected directly from the tails of three sheep flocks (*n* = 335) in Minfeng County, Xinjiang Uygur Autonomous Region, north-western China. Of these, 55 fleas were identified by morphological examination and molecular analysis of four loci (the ribosomal *18S* and *28S* rDNA genes and the mitochondrial genes cytochrome  *c* oxidase subunit II and elongation factor 1-alpha). Eight *Rickettsia*-specific gene fragments originated from seven genes: the 17-kilodalton antigen gene (*17-kDa*), citrate synthase gene (*gltA*), 16S rRNA gene (*rrs*), outer membrane protein A gene (*ompA*), surface cell antigen 1 gene (*sca1*), PS120 protein gene (*gene D*), and outer membrane protein B gene (*ompB*, two fragments), were used to identify the species of *Rickettsia* in 53 fleas. The amplified products were sequenced and included in a phylogenetic analysis to verify the taxonomic identification of the rickettsial agents. Based on morphological and molecular evidence, the flea was identified as *Vermipsylla alakurt*. Nine samples were positive (16.98 %, 9/53) for *Rickettsia* spp. The phylogenetic tree revealed that the rickettsial agents found in *V. alakurt* cluster with Candidatus *Rickettsia barbariae.*

**Conclusions:**

Our study suggests that: (i) *V. alakurt* may serve as a carrier for Candidatus *R. barbariae*; and (ii) Candidatus *R. barbariae*, previously reported in Israel, is the eighth newly discovered validated *Rickettsia* species in China. This finding extends our knowledge of the distribution of Candidatus *R. barbariae* and the profile of carriers, which not only comprise ticks but also fleas.

## Findings

### Background

Fleas (Insecta: Siphonaptera) are small, laterally flattened, wingless, and highly specialised insects [1]. About 2575 species belonging to 16 families and 246 genera have been described, but only a minority is closely associated with humans and other animals [1, 2]. *Vermipsylla* is a genus of the family Vermipsyllidae within the Siphonaptera [3–5]. At least eight species, i.e. *Vermipsylla alakurt* (Kazakhstan, Mongolia, China), *V. asymmetrica* (China), *V. ibexa* (China), *V. minuta* (China), *V. parallela* (China), *V. perplexa* (China, Nepal), *V. quilianensis* (China) and *V. yeae* (China), have been described [6, 7]. *Vermipsylla alakurt* was first identified in China in 1965, in the southern region of Xinjiang Uygur Autonomous Region (XUAR, north-western China) [6]. During December to January, the adult flea is mainly endemic in alpine pastoral areas in XUAR and Qinghai Province (northern China). It infests sheep, yaks and horses, and causes irritation, poor condition, anemia and even death [8–10].

Fleas are mainly blood vessel feeders [11]. The effect of concern of this dietary preference is that fleas themselves are hosts to pathogens, and thus provide a natural avenue for pathogen dispersal [12, 13]. Members of the Rickettsiaceae, such as *Rickettsia typhi* and *R. felis*, are well known as flea-borne pathogens [14]. To the best of our knowledge, little is known about rickettsial agents in *V. alakurt*. In the present study, a molecular investigation was carried out to identify *Rickettsia* spp. in *V. alakurt*.

## Methods

### Collection of fleas and morphological identification

In December 2013, fleas (133 in total) were collected directly from the tails of three sheep flocks (*n* = 335) at Yeyike Town (3300 m above sea level; 36°74ʹ93ʺN, 83°00ʹ26ʺE), Minfeng County, near the Taklimakan Desert, in the southern region of XUAR. The fleas were first identified morphologically. According to an agreement between the Veterinary Research Institute, Xinjiang Academy of Animal Sciences (XAAS) and the School of Medicine, Shihezi University (SU), the fleas were divided into three samples on the basis of their number of morphological differences at the species level. One sample (*n* = 78), belonging to XAAS, was used for full-length mitochondrion sequencing (these data have not been published). The second sample (*n* = 53), belonging to SU, was used for the molecular study of fleas and the detection of flea-borne pathogens. The last sample (*n* = 2, a male and a female) was for morphological identification by the two cooperating units [6, 15].

### Molecular studies on fleas

Total genomic DNA of 53 fleas was extracted from individual specimens using the TIANamp Genomic DNA Kit (TIANGEN, Beijing, China). The DNA of six randomly selected fleas was employed for multi-locus sequence analysis using four genes [18S ribosomal DNA (*18S* rDNA), 28S ribosomal DNA (*28S* rDNA), cytochrome * c* oxidase subunit II (*COII*) and elongation 1-alpha (*EF-1a*)] to examine the phylogenetic relationships within the Siphonaptera. The primers in this study were shown in Table 1. The PCR cycling condition consisted of a pre-PCR step of 95 °C for 5 min, followed by 35 cycles of 95 °C for 40 s, annealing for 50 s at 59.9 °C for amplifying *18S rDNA* and *28S rDNA*, 52.6 °C for *EF-1a* gene, and an extension of 72 °C for 1 min, with a final extension of 72 °C for 10 min. The PCR products were purified using the TIANgel Midi Purification Kit (TIANGEN) and sequenced by Sangon Biotech Co., Ltd (Shanghai, China).

**Table 1:** List of the primers used in the study

Gene	Primer	Sequence (5′–3′)	Reference
*18S* rDNA	18Sai	CCTGAGAAACGGCTACCACATC	[26]
	18S7R	GCATCACAGACCTGTTATTGC	[26]
*28S* rDNA	28SrD3.2a	AGTACGTGAAACCGTTCASGGGT	[26]
	28SrD5b	CCACAGCGCCAGTTCTGCTTAC	[26]
*COII*	A-tLEU	ATGGCAGATTAGTGCAATGG	[27]
	B-tLYS	GTTTAAGAGACCAGTACTTG	[27]
*EF-1a*	EF-1a-F	GGACACAGAGATTTCATCAAGAACA	This study
	EF-1a-R	GCAATGTGRGCHGTGTGGCA	This study
*ompB*	ompB-F	ATTTACAAGCAGGTGGTG	This study
	ompB-R	GCAGTGTTACCGGGATTG	This study
*geneD*	geneD-F	CGGTAACCTAGATACAAGTGA	This study
	geneD-R	TATAAGCTATTGCGTCATCTC	This study

### Detection of rickettsial agents and sequence analysis

For genetic detection of *Rickettsia* spp., six PCR targets were assessed within each sample to determine the presence of *Rickettsiae*: a 434 bp product of the gene encoding the 17 kilodalton antigen (*17-kDa*), 1332 bp of 16S rRNA (*rrs*), 1060 bp of citrate synthase (*gltA*), 488 bp of cell surface antigen 1 (*sca1*), 491 bp of outer membrane protein A (*ompA*), and 812 bp of *ompB*, according to a previous description [16]. To confirm further the presence of rickettsial DNA in *V. alakurt*, two new pairs of primers were designed, based on another region of *ompB* (526 bp) and the PS120-protein-encoding gene (*gene D*; 920 bp) fragment sequences (accession no. GU353186 and EU272188) (see Table 1). PCR conditions consisted of a pre-PCR step of 95 °C for 5 min, followed by 35 cycles of 95 °C for 40 s, annealing for 30 s at 55 °C for amplifying *ompB* and *gene D*, and an extension of 72 °C for 1 min, with a final extension of 72 °C for 10 min. Each PCR assay included a negative control (distilled water instead of flea DNA template) and a positive control (with DNA from *R. raoultii* obtained from wetlands of Ebinur Lake in XUAR) [17]. Purification and sequencing of the positive PCR products were as described above. A phylogenetic tree was constructed using the maximum-likelihood and neighbor-joining algorithms implemented in MEGA 6 software [18].

## Results

**Fig. 1:**
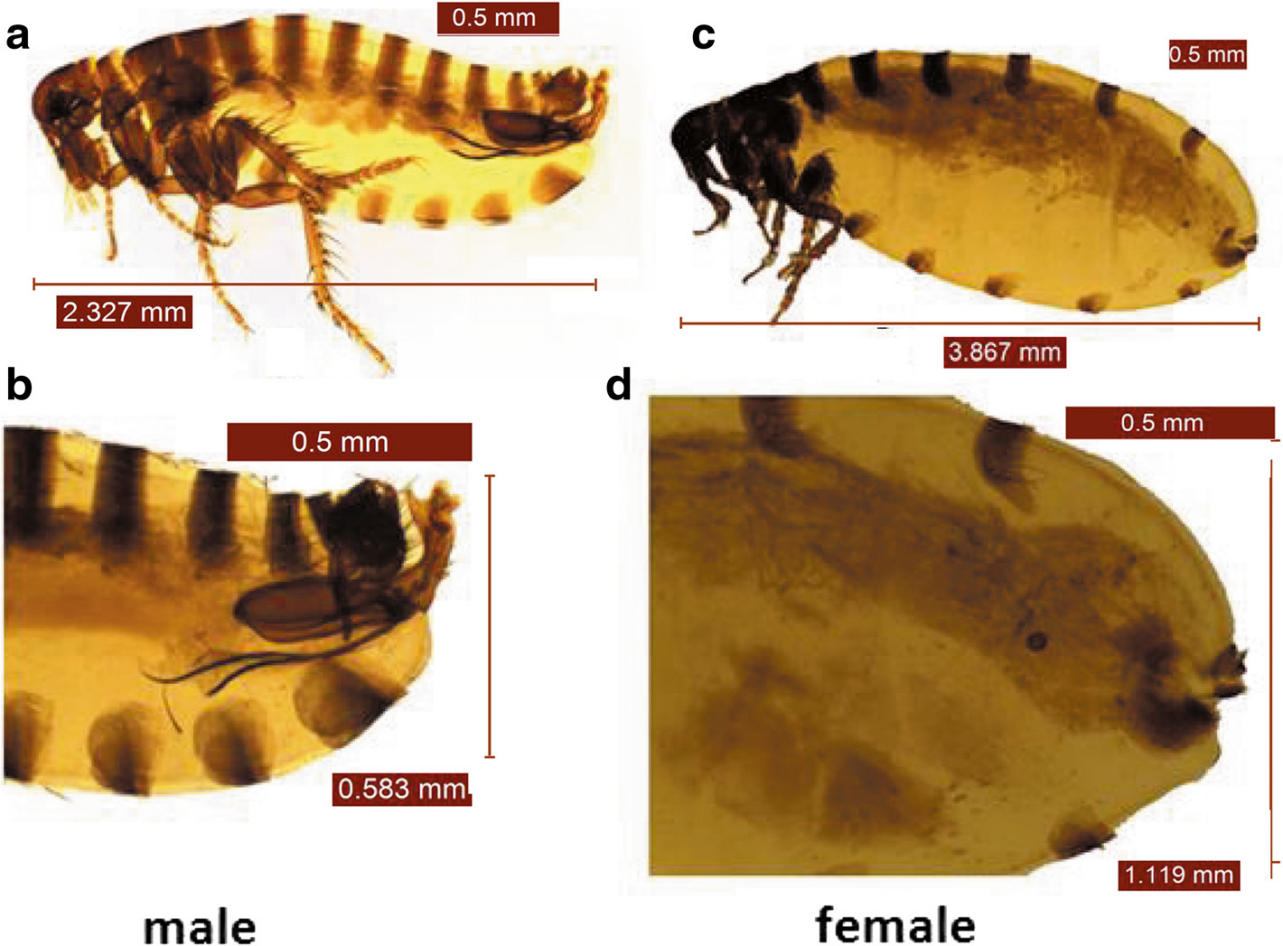
Photomicrographs of *Vermipsylla alakurt*. **a** Male, total view. **b** Male, posterior part of the abdomen. **c** Female, total view. **d** Female, posterior part of the abdomen. The specimens are visualised using a stereomicroscope LEICA EZ4HD equipped with a digital camera

The collected fleas were identified primarily as *V. alakurt* by morphological identification. Their foreheads were smooth and curved without outgrowths. The head of the intromittent organ of the male fleas looks like winter gloves (with the back four fingers held together) (Fig. 1a, b). The head of the spermathecae is ellipsoid, and the tail part is thin and long, with a sausage-like shape (Fig. 1c, d). Data on the four nucleotide sequences (*18S* rDNA, *28S* rDNA, *COII* and *EF-1a*) from the six fleas indicated that the fleas obtained from the sheep had similarity values of 99.22, 98.28, 87.62 and 91.29 %, respectively, with *Chaetopsylla* (*Vermipsyllidae*) (no *V. alakurt* sequence was available in the GenBank database). Four nucleotide sequences from our study have been deposited in the GenBank database (*18S* rDNA: KR297206; *28S* rDNA: KR297207; *COII*: KT193612; and *EF-1a*: KT193613).

**Fig. 2:**
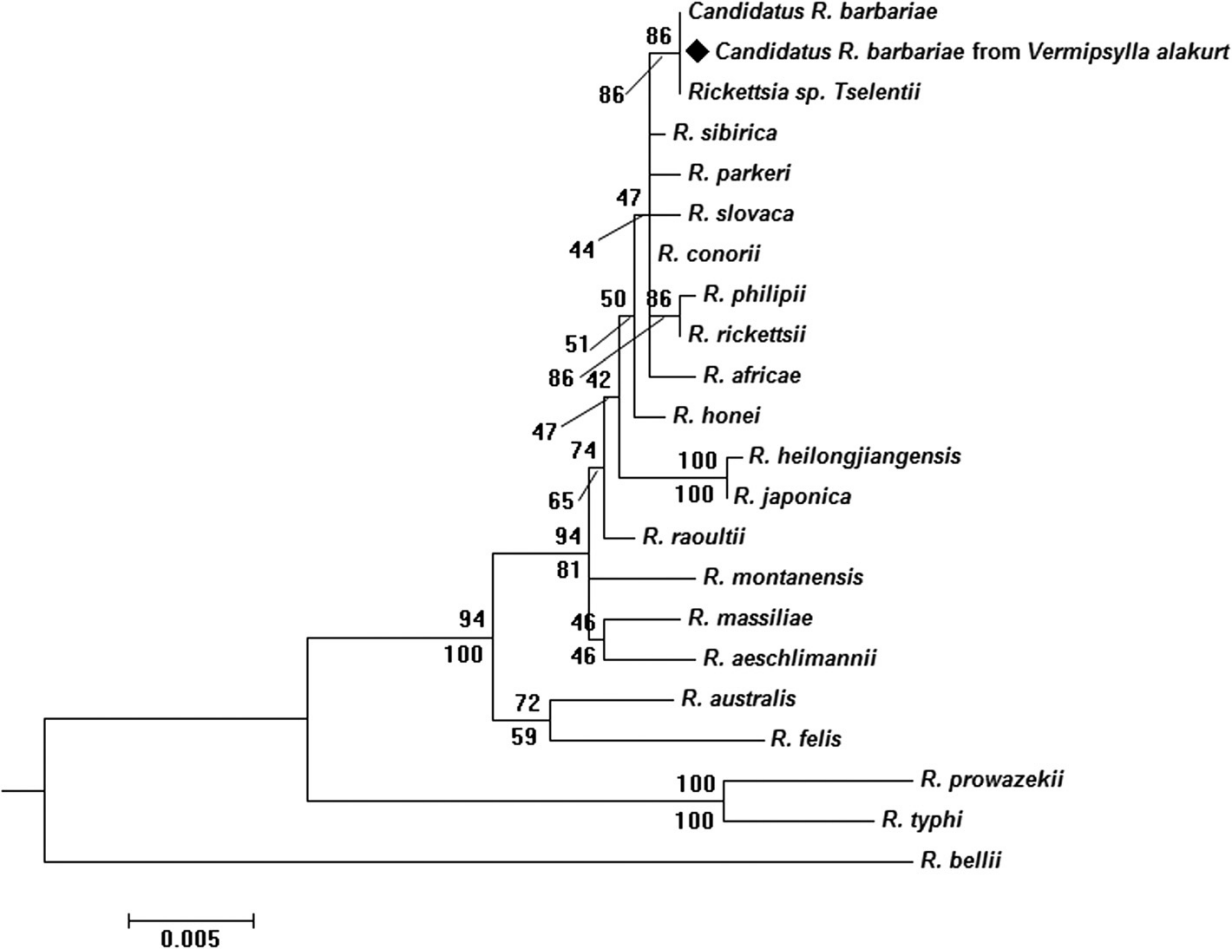
Maximum-likelihood (ML; 1000 bootstrap replicates) and neighbour-joining (NJ; 500 bootstrap replicates) phylogenetic tree of the *17-kDa*-*ompA*-*rrs*-*geneD*-*ompB* constructed with MEGA6, using the sequences of Candidatus *R. barbariae* from *Vermipsylla alakurt* (◆) in this study and sequences from *Rickettsia* species retrieved from the GenBank database. The sequences for *R. bellii* were used as an outgroup. The scale bar represents the inferred substitutions per nucleotide site. The relative support for clades in the tree produced from the ML and NJ analyses are indicated above and below the branches, respectively

Among the 53 flea samples, nine were found to be positive for six rickettsial genetic markers (*17-kDa*, *ompA*, *gltA*, *rrs*, *sca1* and *ompB*). The positive samples were from two sheep flocks. BLAST analysis showed that three of the genetic markers exhibited 99.83 to 100 % similarity with the corresponding sequences of Candidatus *R. barbariae*. The genes *gltA*, *ompB* and *sca1* were exceptions. This was attributed to: (i) the length of the *gltA* sequence from our study was 1080 bp, longer than the available sequences for Candidatus *R. barbariae* in GenBank; (ii) the partial region of *ompB* used in this study (accession no. KT284717) was different from the fragment of the Candidatus *R. barbariae* available in GenBank (accession no. GU353186); (iii) there is no *sca1* reference sequence available from Candidatus *R. barbariae*. To further identify the rickettsial agent in *V. alakurt*, another region of *ompB* (526 bp, accession no. KU645285) and *gene D*, encoding PS120-protein (920 bp, accession no. KU645286), were studied. The BLAST analysis of these sequences showed that they had, respectively, 100 % similarity with Candidatus *R. barbariae* in the loci *ompB* and *gene D*. The detailed sequence information from our study is deposited in the GenBank database (KT284715–KT284718 and KU645283–KU645286). The phylogenetic tree produced from the maximum likelihood and neighbor-joining analyses of the sequence data for five genes (*17-kDa*–*ompA*–*rrs*–*geneD*–*ompB*) revealed that the rickettsial agent in *V. alakurt* clustered with Candidatus *R. barbariae* (Fig. 2).

## Discussion

Candidatus *R. barbariae*, first identified in *Rhipicephalus bursa* ticks from Portugal in 2006 and named *Rickettsia* sp. PoTiRb169 [19], was confirmed and characterised by five genetic markers (*gltA*, *ompA*, *ompB*, *sca4* and *rrs*) in *Rh. turanicus* from Italy in 2008 [20]. Subsequently, the Candidatus *R. barbariae* genotype was respectively detected in *Rh. turanicus* from Cyprus in 2011 and in *Rh. turanicus* and *Rh. sanguineus* from Israel in 2014 [21, 22]. To confirm that the southern region of XUAR might be a natural focus for Candidatus *R. barbariae*, a total of 117 *Rh. turanicus* were collected from sheep during 2013–2014 in six counties around the Taklimakan Desert with the help of Associate Prof. Shi-Wei Wang (College of Animal Science & Technology, Tarim University). Of these, 36 samples (30.76 %) from the six counties were positive for Candidatus *R. barbariae* by seven rickettsial genetic markers (*17-kDa*, *gltA*, *rrs*, *ompA*, *sca1*, *gene D*, and *ompB*). Additionally, we concluded that Candidatus *R. barbariae* may have co-circulated with *R. massiliae* and *R. conorii*, vectored as *Rh. turanicus*, near the Taklimakan Desert before 2013. These findings will be reported in a separate paper.

Candidatus *R. barbariae*, an emerging member of the rickettsial spotted fever group (SFG) [21], has not been reported previously in fleas. Although the vast majority of the SFG rickettsiae are transmitted by ticks, there are exceptions. *Rickettsia africae*, a member of the SFG ordinarily transmitted by ticks, was detected in *Ceratophyllus garei* fleas from passerine birds that had migrated from Africa [23]. Herein, we report the presence of Candidatus *R. barbariae* in *V. alakurt* fleas from sheep in an alpine pastoral area in the north-west of China. This has extended our knowledge of the potential vector spectrum of Candidatus *R. barbariae*.

To date, seven validated SFG *Rickettsia* spp. have been detected in China: *R. heilongjiangii*, *R. sibirica*, *R. raoultii*, *R. slovaca*, *R. felis*, *R. aeschlimannii* and *R. massiliae* [17, 24]*. Rickettsia felis* was first confirmed in ticks (*Rh. sanguineus*), mosquitoes (*Anopheles sinensis* and *Culex pipiens pallens*), lice (*Linognathus setosus*) and fleas (*Ctenocephalides felis*) in China [25]. Here, Candidatus *R. barbariae*, as the eighth validated *Rickettsia* species, was found in China. To the best of our knowledge, this finding extends the area of occurrence for Candidatus *R. barbariae*, and is the second report in Asia*.*

Our findings suggest that the *V. alakurt* parasitising sheep may serve as a carrier for Candidatus *R. barbariae*. In the future, Candidatus *R. barbariae* should be genotypically explored by using genomic sequences or other genetic markers. Addtionally, this rickettisial agent should be further investigated in a wider spectrum in arthropods.

## Conclusions

This is the first report of the presence of Candidatus *R. barbariae* in *V. alakurt* fleas rather than ticks, and of the occurrence of Candidatus *R. barbariae* in China*.* These findings extend our knowledge of the geographical distribution and reservior hosts for Candidatus *R. barbariae*.

## Abbreviations

SFG, spotted fever group; SU, Shihezi University; XAAS, Xinjiang Academy of Animal Sciences; XUAR, Xinjiang Uygur Autonomous Region

## References

[1] Bitam I, Dittmar K, Parola P, Whiting MF, Raoult D (2010). Fleas and flea-borne diseases. Int J Infect Dis.

[2] Whiting MF, Whiting AS, Hastriter MW, Dittmar K (2008). A molecular phylogeny of fleas (Insecta: Siphonaptera): origins and host associations. Cladistics.

[3] Lewis RE, Lewis JH (1994). Siphonaptera of North America north of Mexico: Vermipsyllidae and Rhopalopsyllidae. J Med Entomol.

[4] Lewis RE (1973). Notes on the geographical distribution and host preferences in the order Siphonaptera. 2. Rhopalopsyllidae, Malacopsyllidae and Vermipsyllidae. J Med Entomol.

[5] Lewis RE (1971). A new species of *Chaetopsylla* Kohaut, 1903, infesting pikas in Nepal (Siphonaptera: Vermipsyllidae). J Parasitol.

[6] Liu ZY, Wu HY, Wu FL (1965). On two new species of *Vermipsylla* from west China and a revision of the characters of the genus (Siphonaptera: Vermipsyllidae). Acta Zool Sinica.

[7] ZipcodeZoo. *Vermipsylla alakurt*. http://zipcodezoo.com/index.php/Vermipsylla. Accessed 3 Jan 2011.

[8] Zhao CG, Zhang JS, Meng QL, Qiao J, Shi GJ (2012). Morphological identification of Vermipsyllidae parasiting sheep collected from Tacheng region in Xinjiang. Chin J Prevent Vet Med.

[9] Yao HR, Wang CJ (2008). Investigation on Vermipsyllidae infesting sheep in Hualong County, Qinghai Province. Chin J Vet Med.

[10] Wang GL, Nuer B, Si M, Zhang JC, Xu XJ, Aimaier Y (2000). Outbreak of Vermipsyllidae infesting livestock in Minfeng County, Xinjiang Uygur Autonomous Region. Chin J Vet Parasito.

[11] Vaughan JA, Thomas RE, Silver GM, Wisnewski N, Azad AF (1998). Quantitation of cat immunoglobulins in the hemolymph of cat fleas (Siphonaptera: Pulicidae) after feeding on blood. J Med Entomol.

[12] Eisen RJ, Gage KL (2012). Transmission of flea-borne zoonotic agents. Annu Rev Entomol.

[13] McElroy KM, Blagburn BL, Breitschwerdt EB, Mead PS, McQuiston JH (2010). Flea-associated zoonotic diseases of cats in the USA: bartonellosis, flea-borne rickettsioses, and plague. Trends Parasitol.

[14] Leulmi H, Socolovschi C, Laudisoit A, Houemenou G, Davoust B (2014). Detection of *Rickettsia felis*, *Rickettsia typhi*, Bartonella Species and *Yersinia pestis* in Fleas (Siphonaptera) from Africa. PLoS Negl Trop Dis.

[15] Wang Y, Wang G, Cong PQ, Yu YW (2013). Study on lifecycle of *Vermipsylla alakurt*. Shandong J Anim sci vet Med.

[16] Anstead CA, Chilton NB (2013). A novel *Rickettsia* species detected in vole ticks (*Ixodes angustus*) from Western Canada. Appl Environ Microbiol.

[17] Guo LP, Mu LM, Xu J, Jiang SH, Wang AD (2015). *Rickettsia raoultii* in *Haemaphysalis erinacei* from marbled polecats, China-Kazakhstan border. Parasit Vectors.

[18] Tamura K, Stecher G, Peterson D, Filipski A, Kumar S (2013). MEGA6: molecular evolutionary genetics analysis version 6.0. Mol Biol Evol.

[19] de Sousa R, Barata C, Vitorino L, Santos-Silva M, Carrapato C, Torgal J (2006). *Rickettsia sibirica* isolation from a patient and detection in ticks, Portugal. Emerg Infect Dis.

[20] Mura A, Masala G, Tola S, Satta G, Fois F, Piras P (2008). First direct detection of rickettsial pathogens and a new rickettsia, ‘*Candidatus Rickettsia barbariae*’, in ticks from Sardinia, Italy. Clin Microbiol Infect.

[21] Chochlakis D, Ioannou I, Sandalakis V, Dimitriou T, Kassinis N (2012). Spotted fever group Rickettsiae in ticks in Cyprus. Microb Ecol.

[22] Waner T, Keysary A, Eremeeva ME, Din AB, Mumcuoglu KY (2014). *Rickettsia africae* and Candidatus *Rickettsia barbariae* in ticks in Israel. Am J Trop Med Hyg.

[23] Sekeyová Z, Mediannikov O, Roux V, Subramanian G, Spitalská E (2012). Identification of *Rickettsia africae* and *Wolbachia* sp. in *Ceratophyllus garei* fleas from passerine birds migrated from Africa. Vector Borne Zoonotic Dis.

[24] Wei QQ, Guo LP, Wang AD, Mu LM, Zhang K (2015). The first detection of *Rickettsia aeschlimannii* and *Rickettsia massiliae* in *Rhipicephalus turanicus* ticks, in northwest China. Parasit Vectors.

[25] Zhang J, Lu G, Kelly P, Zhang Z, Wei L (2014). First report of *Rickettsia felis* in China. BMC Infect Dis.

[26] Whiting MF (2002). Mecoptera is paraphyletic: multiple genes and phylogeny of Mecoptera and Siphonaptera. Zool Scr.

[27] Maekawa K, Kitade O, Matsumoto T (1999). Molecular phylogeny of orthopteroid insects based on the mitochondrial cytochrome oxidase II gene. Zoologicalence.

